# The role of KDR in intrauterine adhesions may involve the TGF-β1/Smads signaling pathway

**DOI:** 10.1590/1414-431X20198324

**Published:** 2019-10-07

**Authors:** Jian Xia Chen, Xi Juan Yi, Pei Ling Gu, Shan Xia Gao

**Affiliations:** Department of Reproductive Medicine, Linyi Central Hospital, Linyi City, Shandong, China

**Keywords:** KDR, Intrauterine adhesions, TGF-β1/Smads, MMP9, VEGF

## Abstract

The aim of this study was to investigate the role of kinase-insert domain-containing receptor (KDR) in intrauterine adhesions (IUA) and its mechanism. The Case group consisted of 92 patients diagnosed with IUA, and the Control group included 86 patients with uterine septum who had normal endometrium verified with an uteroscope. In addition, 50 rats were randomly assigned into Control, Sham, Model, NC-siRNA, and KDR-siRNA groups. Rats in the Model, NC-siRNA, and KDR-siRNA groups were induced by uterine curettage and lipopolysaccharide (LPS) treatment to establish the IUA model. Then, immunohistochemistry was applied for detection of VEGF and KDR expression, HE staining was used for observation of the endometrial morphology and gland counting, Masson staining for measurement of the degree of endometrial fibrosis, and qRT-PCR and western blot for the expression of KDR, VEGF, MMP-9, as well as TGF-β1/Smads pathway-related proteins. Compared with the Control group, the mRNA and protein expressions of KDR were significantly higher in IUA endometrial tissues, and the expression of KDR was positively correlated to the severity of IUA. In addition, the injection of si-KDR increased the number of endometrial glands, reduced the area of fibrosis, inhibited mRNA and protein expression of KDR and VEGF, up-regulated the expression of MMP-9 and Smad7, and decreased the expression level of TGF-β1, p-Smad2, p-Smad3, and Smad4 in rats with IUA. Highly-expressed KDR was related to patients' severity of IUA, and silencing KDR may prevent the occurrence and development of IUA via TGF-β1/Smads signaling pathway and up-regulating the expression of MMP-9.

## Introduction

Intrauterine adhesions (IUA), also identified as Asherman's syndrome, are caused by the abnormal healing of the injured endometrial basal layer owing to a consequence of trauma or infection to the endometrium, and the clinical symptoms of IUA include menstrual disturbance (amenorrhea/hypomenorrhea), cyclical abdominal pain, infertility, and recurrent pregnancy loss (1,2). Although the trans-cervical resection of adhesions under the uteroscope is the ideal option for the treatment of IUA, the post-operative recurrence rate is up to 62.5% (3). Presently, the pathogenesis of IUA has not been elucidated, but there are many hypotheses regarding the causes that contribute to IUA, such as fibrosis, nerve reflex, and abnormal differentiation of endometrial stem cells (4), and among them, fibrosis has been the most extensively studied (5). Injury to the basal layer of the endometrium would result in the regeneration of epithelial and interstitial cells, the proliferation of fibroblasts, and the over-accumulation of extracellular matrix, eventually leading to the proliferation of fibrous connective tissues and the formation of cicatrices (5,6). Therefore, considering IUA as a fibrosis-induced disease can provide a good perspective for finding a new therapy.

Vascular endothelial growth factor receptors (VEGFRs), a series of transmembrane receptor tyrosine kinases, could bind VEGF to initiate a signaling cascade that culminates in cellular migration, mitosis, and proliferation, thereby promoting angiogenesis and inducing fibrosis of endometrium (7). To date, five members of VEGFRs have been identified, including VEGFR-1, VEGFR-2, VEGFR-3, Neuropilin-1 (NP-1), and NP-2 (8), while the bioactivity of VEGF was mainly determined through competitively binding to VEGFR-2 (9). VEGFR-2, also known as kinase-insert domain-containing receptor (KDR), is located on human chromosome 4q31.2-q32, and composed of one transmembrane domain, seven extracellular immunoglobulin-like domains, and one cytoplasmic tyrosine kinase domain (10). In particular, KDR can promote mitosis, increase vascular permeability, and is involved in the induction of angiogenesis (11). In recent years, KDR has been demonstrated to be associated with several fibrosis-related diseases. For example, Yan et al. reported that the role of CD147 in promoting liver fibrosis may be related to its impact on the interaction between hepatocytes and sinusoidal endothelial cells through the mediation of VEGF-A/KDR signaling pathway (12). In addition, fibrosis was closely related to the pathogenesis of IUA (5). Moreover, in the study by Kim et al. (13), KDR was found to be able to affect the formation of adhesions and the experiment on a murine model proved that sunitinib, a KDR antagonist, can remarkably inhibit adhesion formation. Nevertheless, the involvement of KDR in the development and progression of IUA is still unclear.

Therefore, we detected the KDR expression in endometrial tissues with or without IUA, and further analyzed the expression of TGF-β1/Smads signaling pathway by the injection of si-KDR into the uterus after establishing the IUA rat model to explore the mechanism of KDR in IUA formation in depth.

## Material and Methods

### Ethics statement

The experiment in this study was approved by the Ethics Committee of our hospital and performed in obedience to the Helsinki declaration (14). All specimens were obtained with the informed consent of each patient who volunteered to participate in the clinical trial. All animal experiments were in line with the principles for management and use of local laboratory animals and followed the Guide for the Care and Use of Laboratory Animals published by the National Institutes of Health (15).

### Study subjects

From May 2016 to May 2017, 92 patients diagnosed and confirmed with IUA in our hospital were included as the Case group (age range: 20–50 years; average age: 35.3±4.1 years). Based on the American Fertility Society classification of IUAs (16), there were 38 cases of IUA-I patients, 30 cases of IUA-II patients, and 24 cases of IUA-III patients. All patients were in the proliferative phase of their menstrual cycle, and postoperative specimens pathologically confirmed who had no gynecological endocrine diseases or fibrosis-related diseases/operations. Patients had not used an intrauterine device or hormone drugs within the latest 6 months and had complete clinical data. The Control group consisted of 86 patients who had normal endometrium and regular menstrual cycles but had uterine septum operated with a uteroscope in our hospital.

A small amount of endometrial tissues near the adhesions was obtained from patients in the Case group using a pair of forceps; and the same amount of normal endometrial tissues was taken from those in the Control group. Two specimens were obtained from each patient. One specimen was immediately deposited in RNA-later solution before being preserved in a refrigerator at −80°C within 24 h. The other one was rinsed with normal saline to wash away blood, placed in a container with formalin for 24 h, transferred to a container with sodium azide preservation solution, and preserved in a refrigerator at −4°C.

### Immunohistochemical staining

Sections were dewaxed in xylene, rehydrated through graded concentrations of ethanol, and placed at room temperature for 15 min before washing with phosphate buffered saline (PBS). The blocking solution of normal goat serum was added at room temperature for 20 min, followed by 1 h incubation with KDR (ab184903, Abcam, USA) antibody at 37°C, washing with PBS, and another 1 h of incubation with the secondary antibody at room temperature. After that, tissue sections were washed again with PBS and developed with diaminobenzidine (DAB) to observe the coloration under a microscope. Next, hematoxylin was used for 2 min of re-dyeing, followed by routine dehydration, hyalinization, mounting, and microscopic examination. The statistical analysis of positive rate was conducted based on the classification by Fromowitz et al. (17): 0 point was regarded as negative (−); 2–3 points, as weakly positive (+); 4–5 points as positive (++); and 6–8 points as strongly positive (+++); (−) and (+) meant low expression, while (++) and (+++) meant high expression.

### Establishment of IUA model rats

Fifty female non-pregnant Wistar rats with a weight about 180–200 g were randomly classified into five groups: Control group, Sham group, Model group, NC-siRNA group, and KDR-siRNA group, with 10 rats in each group. Except for those in the Control group, all rats in other groups received dual (mechanical and infectious) injury using uterine curettage and lipopolysaccharide (LPS) surgical suture to establish the model of IUAs (18). In brief, the rats were anesthetized by ether and cut on the skin about 0.5 cm over the urethra. The endometrial lining of the upper uterus was scraped with a mini-endometrial curette, and the LPS surgical suture (10 mg/L, derived from *Escherichiacoli 055: B5*; Sigma, USA) was inserted through the uterine cavity after the curettage using a round surgical needle. Then, the plasmids (Shanghai GenePharma Co., Ltd., China) were immediately injected into five different sites of the uterus (18). In addition, rats in the Control group and Model group were injected with 200 μL physiological saline; those in the NC-siRNA group and the KDR-siRNA group with 2.5 nmol NC-siRNA and KDR interference plasmid dissolved in 200 μL physiological saline, respectively. Four days later, rats in the NC-siRNA group and KDR-siRNA group were given the same dose of NC-siRNA and KDR-siRNA, respectively, while the control group did not receive any treatment.

### Specimen collection

The animals were killed by cervical dislocation and their bilateral uterine tissues were collected at the end of the second estrous cycle (9 days), showing highly fibrous adhesions according to the record of Li et al. (18). Next, four samples were taken from each uterine tissue. TRIzol reagent was added to one uterine tissue sample to extract RNA for qRT-PCR detection; one was fixed in 4% paraformaldehyde and embedded with paraffin to make paraffin sections for HE staining, Masson staining, and immunohistochemistry; one was preserved at −80°C for later protein extraction and western blotting; and the remaining uterine tissues were placed in a liquid nitrogen container for later use.

### HE staining and Masson's trichrome staining

The endometrial tissues of rats were fixed for 24 h in Davidson's solution before the routine procedures of dehydration, hyalinization, wax dipping, and paraffin embedding. Next, the ultra-thin semiautomatic microtome (Shandon 325, UK) was used to slice ten 3-μm-thick serial sections, which were baked at 50°C for 1 h, stained with hematoxylin and eosin, and observed under a microscope (Leica DMLB2, Germany) to evaluate the pathological changes of endometrial tissues. Then, conventional Masson's trichrome staining (Weigert's iron hematoxylin, Ponceau, and Aniline blue) was performed on tissues. After that, five visual fields were chosen randomly from the stained tissues under the high-power lens, and the software Image Pro Plus 6.0 (Media Cybernetics, USA) was used for the analysis of pictures of pathological tissues. The number of endometrial glands and degree of endometrial fibrosis were microscopically evaluated as described previously (19).

### qRT-PCR

The endometrial tissues were ground with normal saline, and the RNA extraction kit (Omega, USA) was used to extract the total RNA of each tissue sample. RNA purity and concentration were determined by the UV spectrophotometer (UV-1800, Japan) and observed for integrity by agarose gel electrophoresis. The primers of VEGF and KDR were designed using the software Primer 5.0 (Premier Biosoft, USA) and synthesized by Sangon Biotech Co., Ltd. (China). cDNA was generated through the reverse transcription of the total RNA using Primescript^TM^ RT reagent Kit (Takara, Japan). The reverse transcription system was 10 μL in volume, and the conditions for reaction were as follows: 16°C for 30 min, 42°C for 30 min, and 85°C for 10 min. The SYBR^®^ premix Ex Taq TMPCR kit (Takara Biotechnology Co., Ltd., China) was used for qRT-PCR detection and the reaction conditions were: pre-denaturation for 2 min at 95°C, and 40 cycles of denaturation for 5 s at 95°C, annealing for 5 s at 60°C, and extending for 30 s at 72°C. The expression of mRNA was calculated using 2^-△△Ct^ with GAPDH as the internal reference gene.

### Western blot

The endometrial tissues of each group were added with lysate, homogenized, and centrifuged at 1,200 *g* for 15 min at 4^o^C. Next, the upper supernatant was collected for SDS-PAGE electrophoresis. The proteins were electrically transferred to the nitrocellulose membrane for blocking with 5% skim milk-PBS solution at room temperature. One hour later, the primary antibodies were added for overnight incubation at 4°C, including VEGF (ab46154, 1 µg/mL), KDR (ab11939, 1 µg/mL), TGF-β1 (ab92486, 4 µg/mL), pSmad3 (ab52903, 1/2000), Smad3 (ab40854, 1/5000), pSmad2 (ab53100, 1/1000), Smad2 (ab40855, 1/2000), Smad4 (ab40759, 1/5000), Smad7 (ab216428, 1/300), MMP-9(ab38898, 1/1000), and β-actin (ab8226, 1 µg/mL) (purchased from Abcam, USA). The secondary antibody crosslinked with HRP was added at room temperature for 1 h after the membrane was rinsed with PBS buffer three times. Blots were then developed by enhanced chemiluminescence (ECL) detection. With β-actin as the loading control, the gray value ratio of target band to reference band was regarded as the relative expression level of proteins.

### Statistical analysis

All data were analyzed using the statistical software SPSS 20.0 (IBM, USA). The measurement data are reported as means±SD, with the difference between two groups compared by least significant difference (LSD) test, or among multiple groups tested by one-way ANOVA. Enumeration data were analyzed and tested by chi-squared test or Fisher's exact test, and the relationship between variables was assessed by Spearman's correlation analysis. A P value less than 0.05 was regarded as statistically significant.

## Results

### Expression of KDR in the endometrium tissue of IUA patients and its relationship with severity of IUA

**Figure 1:**
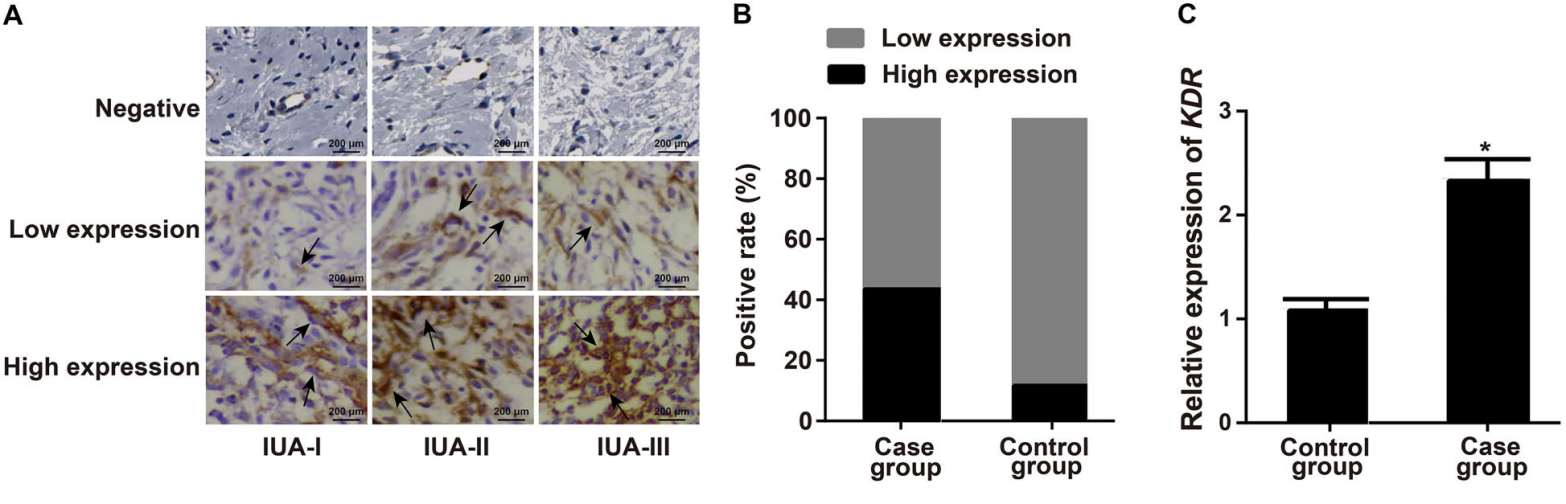
Kinase-insert domain-containing receptor (KDR) expression in the endometrium tissues of patients from the Case group and the Control group. **A**, Protein expression of KDR in the endometrium tissues of intrauterine adhesions (IUA) patients detected by immunohistochemical staining. The localization of KDR (black arrows) was observed in IUA-I, IUA-II, and IUA-III patients. **B**, Positive rate of KDR expression in the endometrium tissues of the Case group and the Control group. **C**, Relative expression of *KDR* in endometrium tissues of the Case group and the Control group determined by qRT-PCR. Data are reported as means±SD. *P<0.05 compared with the Control group (least significant difference test).

The KDR protein identified by immunohistochemical staining (Figure 1A) was mainly localized in the membrane and cytoplasm of endometrial squamous epithelial cells. As exhibited in Figure 1B, the positive rate of KDR expression in the endometrial tissues of IUA from the Case group [43.48% (40/92)] was significantly higher than the Control group [11.63% (10/86)] (P<0.05). qRT-PCR confirmed that the relative mRNA expression of KDR was statistically higher in IUA patients compared to normal controls (P<0.05, Figure 1C). Moreover, the positive expression rate of KDR and its mRNA increased correspondingly with the severity of IUA according to the Spearman correlation analysis (both P<0.05, Table 1).

**Table 1:** Association of the positive expression of KDR and its mRNA with the severity of intrauterine adhesions (IUA).

Severity of IUA	N	KDR positive expression				Positive expression rate	r	P	KDR mRNA		
		-	+	++	+++				Mean±SD	r	P
IUA-I	38	21	12	4	1	5.43%			2.177±0.140		
IUA-II	30	4	10	9	7	17.39%			2.384±0.182		
IUA-III	24	2	3	9	10	20.65%	0.581	<0.001	2.505±0.163	0.692	<0.001

Classification according to Fromowitz et al. 17: 0 point: negative (−); 2–3 points: weakly positive (+); 4–5 points: positive (++); 6–8 points: strongly positive (+++); (−) and (+): low expression; (++) and (+++): high expression. KDR: kinase-insert domain-containing receptor.

### Morphological changes of the endometrium

**Figure 2:**
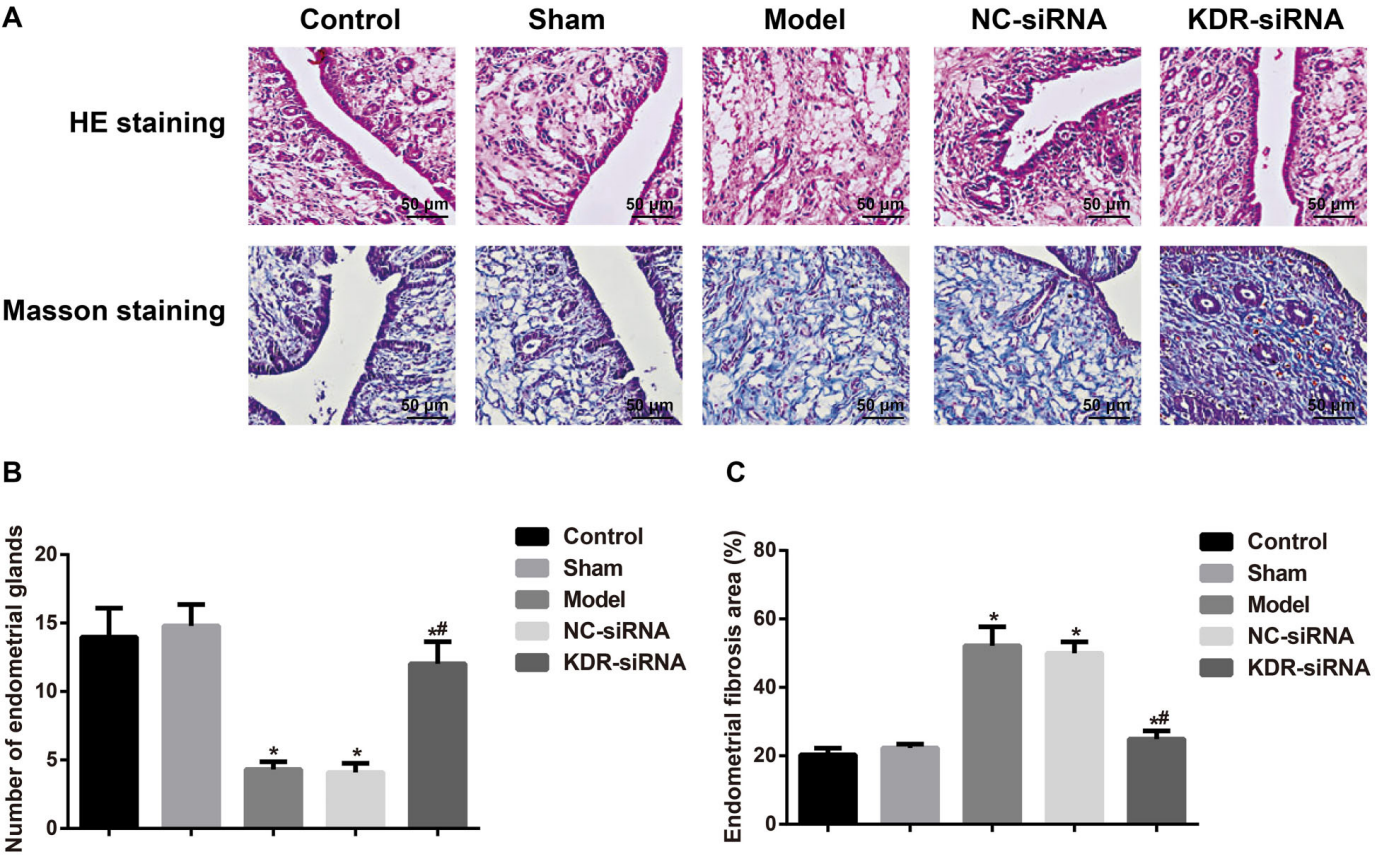
**A**, Morphological changes of endometrium tissues observed by HE staining and Masson staining on the 9th day after model establishment (×400; bar: 50 μm). **B**, Comparison of the number of endometrial glands in each group. **C**, Comparison of endometrial fibrosis area in each group. Data are reported as means±SD.*P<0.05 compared with the Control group and the Sham group; ^#^P<0.05 compared with the Model group and the NC-siRNA group (ANOVA). KDR: kinase-insert domain-containing receptor.

The endometrial cavity surface of the Control and Sham groups was covered by columnar epithelium with abundant endometrial glands arranged in round or oval shape observed by HE staining. Masson staining showed a few stromal collagen fibers and wavily arrayed. Compared with the Control and Sham groups, the rats in the other groups showed different degrees of IUA, a disappearance of endometrial epithelium, and a reduction of glands with tissue hyperplasia, as well as an increase of collagen fibers (Figure 2). Compared with the Model group, the rats in the KDR-siRNA group had an increased number of endometrial glands, which were fewer in the fibrosis area. Those in the NC-siRNA group did not have an observable difference in these two aspects.

### Comparison of expressions of KDR and VEGF

**Figure 3:**
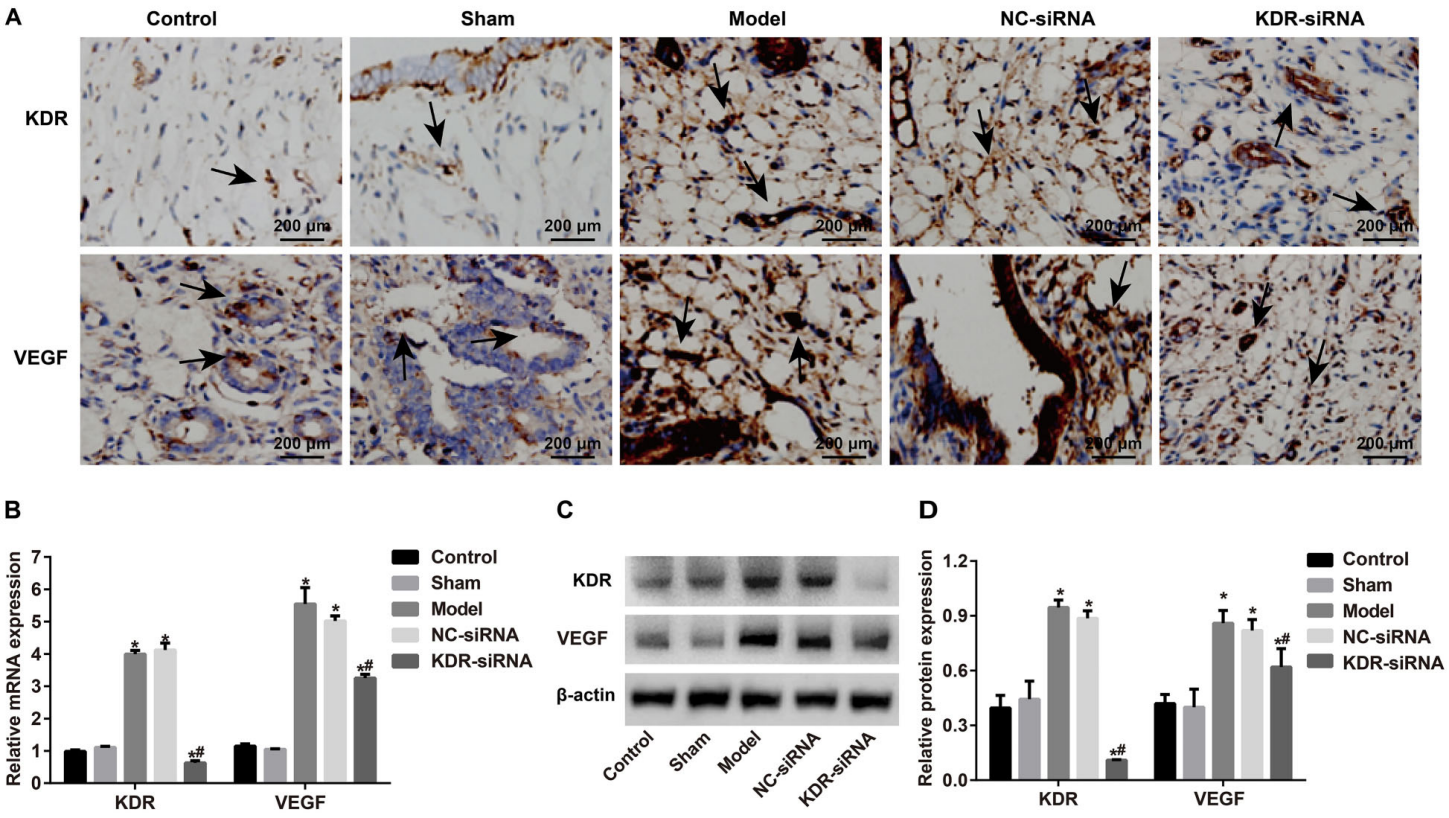
Comparison of expressions of kinase-insert domain-containing receptor (KDR) and vascular endothelial growth factor (VEGF) in the endometrial tissues of rats in each group at the 9th day after model establishment. **A**, Representative localization of KDR and VEGF (black arrow) in the endometrium tissues of rats in each group detected by immunohistochemical staining. **B**, Relative mRNA expressions of KDR and VEGF in endometrium tissues of rats in each group determined by qRT-PCR. **C** and **D**, Protein expression of KDR and VEGF in endometrium tissues of rats in each group detected by western blot. Data are reported as means±SD. *P<0.05 compared with the Control group and the Sham group; ^#^P<0.05 compared with the Model group and the NC-siRNA group (ANOVA).

Compared with the Control and Sham groups, the expressions of both KDR and VEGF at the mRNA and protein levels were enhanced in the endometrial tissues of rats in the Model group (all P<0.05). However, those rats in the KDR-siRNA group presented down-regulated KDR and up-regulated VEGF. In addition, the expressions of KDR and VEGF were significantly lower in the KDR-siRNA group than in the Model group and NC-siRNA group (both P<0.05, Figure 3).

### Expression levels of TGF-β1/Smads pathway-related proteins

**Figure 4:**
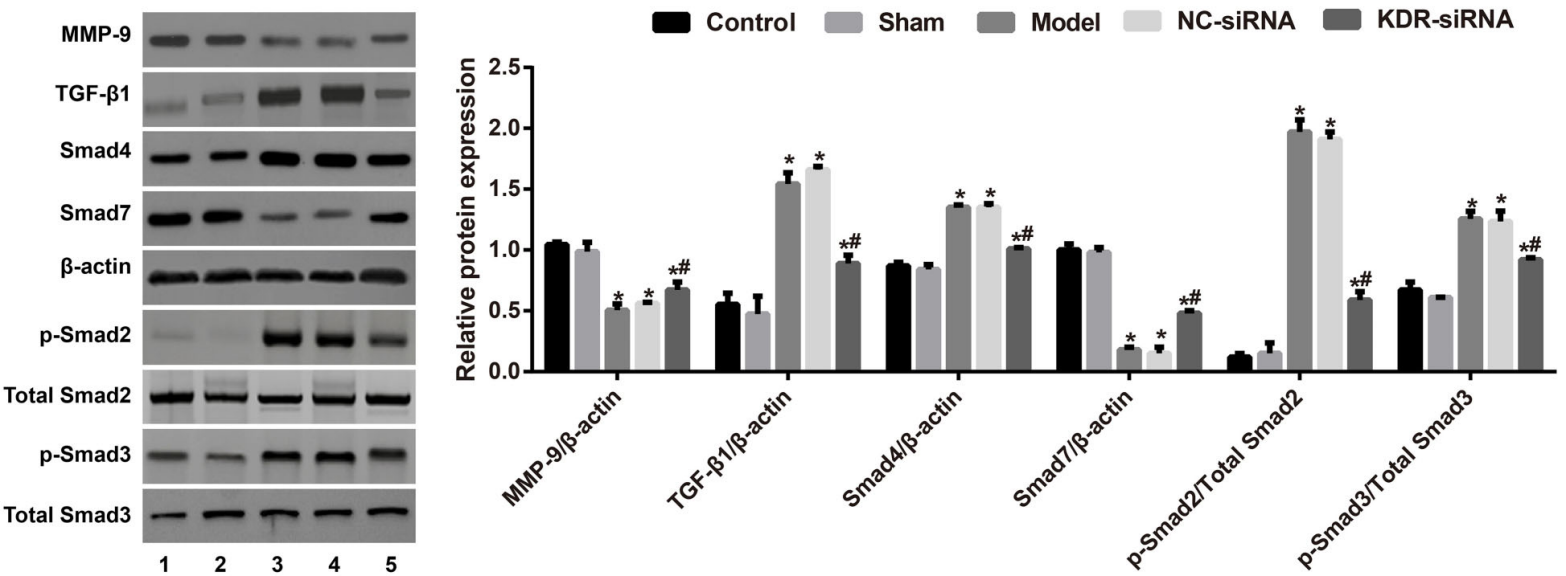
The expression levels of MMP-9 and TGF-β1/Smads pathway-related proteins in the endometrial tissues of rats in each group. 1, Control group; 2, Sham group; 3, Model group; 4, NC-siRNA group; 5, KDR-siRNA group. Data are reported as means±SD. *P<0.05 compared with the Control group and the Sham group; ^#^P<0.05 compared with the Model group and the NC-siRNA group (ANOVA). KDR: kinase-insert domain-containing receptor.

As displayed by western blot in Figure 4, the expressions of TGF-β1, p-Smad2, p-Smad3, and Smad4 were increased, while the expressions of MMP-9 and Smad7 were decreased in the endometrial tissues of rats in the Model group (all P<0.05). Compared with the Model group, the NC-siRNA group showed no changes in any index (all P>0.05); however, the rats in the KDR-siRNA group had an increase in MMP-9 and Smad7 levels, and a decrease in the expression levels of TGF-β1, p-Smad2, p-Smad3, and Smad4 in the endometrial tissue (all P<0.05). Importantly, there were no significant differences with respect to total-Smad2 and total-Smad3 among all groups of rats (all P>0.05).

## Discussion

Currently, the clinically therapy for IUA is effective for the restoration the uterine shape, but the restoration of the physiological function of the uterus is not ideal (20). Thus, it is of great significance to study the molecular mechanisms of the pathogenesis of IUA. In the present research, the mRNA and protein expression of KDR was statistically higher in the endometrium of IUA patients than that of controls, and the expression levels were positively correlated to the severity of IUA. The possible reason may be that KDR, acting as the major medium for the growth and permeability of endothelial cell, can promote the formation of adhesions through the regulation of angiogenesis (13). Cassidy et al. (21) also observed that the KDR mRNA levels in the peritoneal adhesion tissue were reduced by nearly 50% after a single intraoperative dose of valproic acid (VPA), with the reduction of adhesiogenic substrates extravasating into the peritoneum, thereby providing a new perspective for the clinical prevention and treatment of adhesions. Additionally, the binding of VEGF to the KDR has a great impact on the breakdown of cell junctions, which is closely involved in the regulation of vascular permeability (22). These findings suggest that KDR can take part in the occurrence of adhesions partly because of its function in binding to VEGF.

To further investigate the mechanism of KDR in affecting IUA, we constructed the IUA rat model using phenol mucilage and injected KDR-siRNA or NC-siRNA into the uterus of rats. At the 9th day after model establishment, we found increased levels of KDR and VEGF, as well as an enlarged fibrosis area in the rats from the NC-siRNA group, which further confirmed the clinical result and the hypotheses mentioned previously. On the contrary, the elevation of gland number and the reduction of fibrosis area in the endometrium were discovered in the IUA rats treated with si-KDR. Interestingly, there was evidence of a close relationship between VEGF-induced cell proliferation and adhesions and the KDR downstream signaling pathways (23), and our study demonstrated that silencing KDR can contribute to the down-regulation of VEGF, which was consistent with the finding by Aesoy et al. (24), indicating that VEGF may interact with KDR in the endometrium of IUA patients. To date, accumulating reports have shown that VEGF can participate in the occurrence and progression of IUA. For example, the significantly higher expression level of VEGF was reported in the study by Chen et al. (25) in IUA patients than those in the normal controls. Notably, the expression of endometrial VEGF in patients with serious IUA was extremely higher than those with mild and moderate IUA, as suggested by Han et al. (26). Moreover, Chaudhary and his group demonstrated that inhibition of the signaling pathways modulated by VEGF significantly attenuated fibrosis-associated diseases (27). Additionally, Chatterjee et al. (28) revealed that the deficiency of Jam-A would result in the up-regulation of KDR and an accumulation of fibroblasts near the wound area, and eventually cause the formation of cicatrices, showing that KDR can interact with VEGF to induce endometrial fibrosis, and thus promoting the occurrence and development of IUA.

On the other hand, among various fibrosis-related cytokines, TGF-β, a pleiotropic cytokine, was well-accepted as the promoter of fibrotic lesions (29), which consisted of five subtypes, including TGF-β1 (30). Meanwhile, the highly homologous TGF-β receptor-regulated Smads (R-Smads), such as Smad2/Smad3, are the first signaling molecules transmitted under the TGF-β1 signal and the intracellular kinase substrate of TGF-β1 receptor (31). Many studies have indicated that TGF-β1/Smad2/3 may be the signaling proteins closely related to the formation and progression of tissue fibrosis (32). The persistent high concentration of TGF-β1 was the main cause of cicatrices formation in the repair of endometrial trauma in a previous study regarding IUA (20). Phosphorylated Smad2/3could combine with Smad4 to form a complex in the nucleus, resulting in enhanced expression of intracellular and extracellular fibrogenic proteins (33). Also, TGF-β1/Smad3/Smad7 pathway was found to play an important role in the development and progression of IUA (34). In our study, when the IUA rats were treated with the KDR-siRNA, the expressions of TGF-β1, p-Smad2, p-Smad3, and Smad4 in the endometrial tissues were lowered, but Smad7 expression was increased, indicating that silencing KDR can inhibit the IUA presence via TGF-β1/Smads signaling pathway. Similarly, Ou et al. (35) also demonstrated that the early administration of KDR antagonist SU5416 could inhibit the deposition of pulmonary collagen, the abnormal pathologic proliferation of fibrous tissues, and the activation of TGF-beta1/Smad3 pathway in bleomycin-treated rats. Furthermore, some cytokines can induce the expression of TGF-β1 to downregulate MMPs under the pathological status, resulting in the accumulation of extracellular matrix and the formation of tissue fibrosis (36). As a gelatinase in MMPs, MMP-9 mainly induces the degradation of denatured collagen and basal lamina collagen IV (37). According to the results of a previous study, the administration of Fukang oral liquid had an effect on preventing IUA possibly by regulating the expressions of TGF-β1 and MMP-9 in the endometrium (38). Our finding also demonstrated a significant decrease of MMP-9 in the IUA rats from the KDR-siRNA group compared with the negative controls, which provided a possible explanation for KDR action in preventing the fibrosis of endometrial tissues and eventually attenuating IUA through the regulation of the expression of TGF-β1/Smad2/3 pathway and MMP-9.

Collectively, increased KDR was found in the endometrium of IUA patients, which was positively related to IUA severity. However, after silencing KDR, the expression of KDR and VEGF declined and the TGF-β1/Smads pathway was greatly inhibited, with the elevation of MMP-9 and the reduction of fibrosis, thus decreasing the occurrence and development of IUA.
